# Clinical outcomes of transoral videolaryngoscopic surgery for hypopharyngeal and supraglottic cancer

**DOI:** 10.1186/s12885-017-3396-0

**Published:** 2017-06-26

**Authors:** Yorihisa Imanishi, Hiroyuki Ozawa, Koji Sakamoto, Ryoichi Fujii, Seiji Shigetomi, Noboru Habu, Kuninori Otsuka, Yoichiro Sato, Yoshihiro Watanabe, Mariko Sekimizu, Fumihiro Ito, Toshiki Tomita, Kaoru Ogawa

**Affiliations:** 1 Department of Otorhinolaryngology–Head and Neck Surgery, Keio University School of Medicine, 35 Shinanomachi, Shinjuku, Tokyo, 160-8582 Japan; 2 Department of Otorhinolaryngology, Kawasaki Municipal Kawasaki Hospital, Kawasaki, Kanagawa 210-0013 Japan; 3 Department of Otorhinolaryngology, Saiseikai Utsunomiya Hospital, Utsunomiya, Tochigi, 321-0974 Japan; 4 Department of Otorhinolaryngology, Saiseikai Yokohamashi Nanbu Hospital, Yokohama, Kanagawa 234-0054 Japan; 5 Department of Otorhinolaryngology, Yokohama Municipal Citizen’s Hospital, Yokohama, Kanagawa 240-8555 Japan; 6 Department of Otorhinolaryngology, Kyosai Tachikawa Hospital, Tachikawa, Tokyo, 190-0022 Japan; 7 Department of Otorhinolaryngology, Saiseikai Yokohamashi Tobu Hospital, Yokohama, Kanagawa 230-8765 Japan

**Keywords:** Transoral videolaryngoscopic surgery (TOVS), Hypopharyngeal cancer, Supraglottic cancer, Organ-function preservation, Long-term treatment outcomes, Survival, Prognostic factor

## Abstract

**Background:**

Transoral videolaryngoscopic surgery (TOVS) was developed as a new distinct surgical procedure for hypopharyngeal cancer (HPC) and supraglottic cancer (SGC) staged at up to T3. However, long-term treatment outcomes of TOVS remain to be validated.

**Methods:**

Under a straight broad intraluminal view provided by combined use of a distending laryngoscope and a videolaryngoscope, we performed en bloc tumor resection via direct bimanual handling of the ready-made straight-form surgical instruments and devices. We retrospectively analyzed functional and oncologic outcomes of 72 patients with HPC (*n* = 58) or SGC (*n* = 14) whose minimum follow-up was 24 months or until death.

**Results:**

The cohort comprised nine patients of Tis, 23 of T1, 33 of T2, and 7 of T3. Among 36 patients (50%) who underwent neck dissection simultaneously, all but one were pathologically node-positive. Twelve patients underwent postoperative concurrent chemoradiation (CCRT) as adjuvant treatment, and another four patients underwent radiation or CCRT for second or later primary cancer. The endotracheal tube was removed in an operation room in all but two patients who underwent temporary tracheostomy. Pharyngeal fistula was formed transiently in two patients. The median time until patients resumed oral intake and could take a soft meal was 2 and 5 days, respectively. Eventually, 69 patients (96%) took normal meals. The 5-year cause-specific survival (CSS), overall survival (OS), larynx-preserved CSS, and loco-regional controlled CSS were 87.3%, 77.9%, 86.0%, and 88.0%, respectively. Multivariate analysis revealed N2-3 as an independent prognostic factor in both CSS (hazard ratio [HR] = 25.51, *P* = 0.008) and OS (HR = 4.90, *P* = 0.022), which indirectly reflected higher risk of delayed distant metastasis.

**Conclusions:**

Considering its sound functional and oncological outcomes with various practical advantages, TOVS can be a dependable, less invasive, and cost-effective surgical option of an organ-function preservation strategy for HPC and SGC.

## Background

Hypopharyngeal cancer (HPC) affects 0.8–1.3 per 100,000 persons per year in the US, accounting for approximately 6.5% of all head and neck squamous cell carcinomas (SCC) [1]. Unfortunately, prognosis of the patients with HPC reportedly remains the worst among all head and neck subsites, largely because the vast majority of the patients present at a locally advanced stage [2]. Since radical resection for HPC inevitably impairs laryngopharyngeal function, such as vocalization, swallowing, and breathing through the natural airway, organ-function preservation strategies have been increasingly developed, even for treatment of HPC, since the 1990s [3, 4].

Practically, there are three major options that meet the concept of organ-function preservation in the laryngopharyngeal region: radiation (RT) or chemoradiation (CRT), open partial pharyngolaryngectomy (PPL), and transoral surgery. RT or CRT has long been representative of non-surgical treatments, and concurrent CRT (CCRT), in particular, has been recognized as one of the standard therapies for advanced-staged HPC and supraglottic cancer (SGC) [5–7]. However, intensified CCRT with a high-dose regimen results in severe long-term adverse effects including subsequent loss of function in preserved organs [8–12]. Open PPL has also been established as a surgical organ-function preserving procedure for selected cases of early T-staged HPC and SGC [13–15]. Although both oncological and functional outcomes of open PPL have shown to be eventually satisfactory, the surgical invasiveness associated with external incision, reconstruction procedure, and tracheostomy necessitate cautious postoperative managements and relatively long rehabilitation periods, which may make this procedure less popular.

Transoral surgery has emerged as another therapeutic option for laryngopharyngeal lesions. Because of its less invasiveness compared to CCRT regarding treatment-induced long-term toxicity and to open PPL regarding direct histological damage to the surrounding normal tissues, transoral surgery is expected to be an ideal alternative for the treatment of HPC patients. Traditionally, application of transoral surgery had been confined to early tumors in oral, oropharyngeal (except for tongue base), and glottic regions, because of the anatomically limited visualization and manipulation due to a lack of suitable optical instruments. Technological advancements in microscopic/endoscopic monitoring and surgical supporting devices have enabled development of various transoral surgical methods that can approach the hypopharyngeal and supraglottic regions, such as transoral laser microsurgery (TLM) using a microscope since the late 1990s [16–22], and more recently, transoral robotic surgery (TORS) using a surgical robot since the late 2000s [23–30].

Besides the above-mentioned procedures, Shiotani et al. have developed a distinct, unique, non-robotic surgical method custom-built for transoral partial pharyngolaryngectomy since the 2000s; this was subsequently renamed “transoral videolaryngoscopic surgery (TOVS)” [31–33]. In this system, combined use of a distending laryngoscope with a rigid endoscope (videolaryngoscope) can provide a broad intraluminal field of view and a wide working space throughout the upper aero-digestive tract, which facilitates en bloc tumor resection via direct bimanual handling and application of the ready-made straight-form surgical instruments and devices. Favorable oncological outcomes and good functional results have been achieved so far by employing TOVS for T1, T2, and selected T3 cancers of the hypopharynx, supraglottis, and oropharynx [32, 33]. However, because it has not been long since this promising method was introduced, the long-term treatment outcome of TOVS remains to be validated.

The aim of this paper was to retrospectively evaluate clinical outcomes of TOVS for a cohort of patients with HPC and SGC in a tertiary referral center.

## Methods

### Indication for TOVS

All patients were staged according to the UICC TNM classification and staging system [34]. TOVS was applied to patients with HPC and/or SGC staged at Tis, T1, T2, and T3 (classified mainly by size criteria) for the curative resection of a primary lesion. Patients with neck lymph node metastasis were also included unless nodal lesions were considered unresectable.

The exclusion criteria were as follows: (1) medical contraindication to general anesthesia; (2) involvement of the thyroid cartilage, cricoid cartilage, or hyoid bone (i.e., T4 tumor); (3) invasion of bilateral arytenoid cartilages; or (4) extension to more than a semi-circumference of the esophageal entrance. Those patients underwent other treatments including RT, CRT, open PPL, total laryngectomy, or total pharyngolaryngectomy.

### Pre-surgical evaluation

**Fig. 1:**
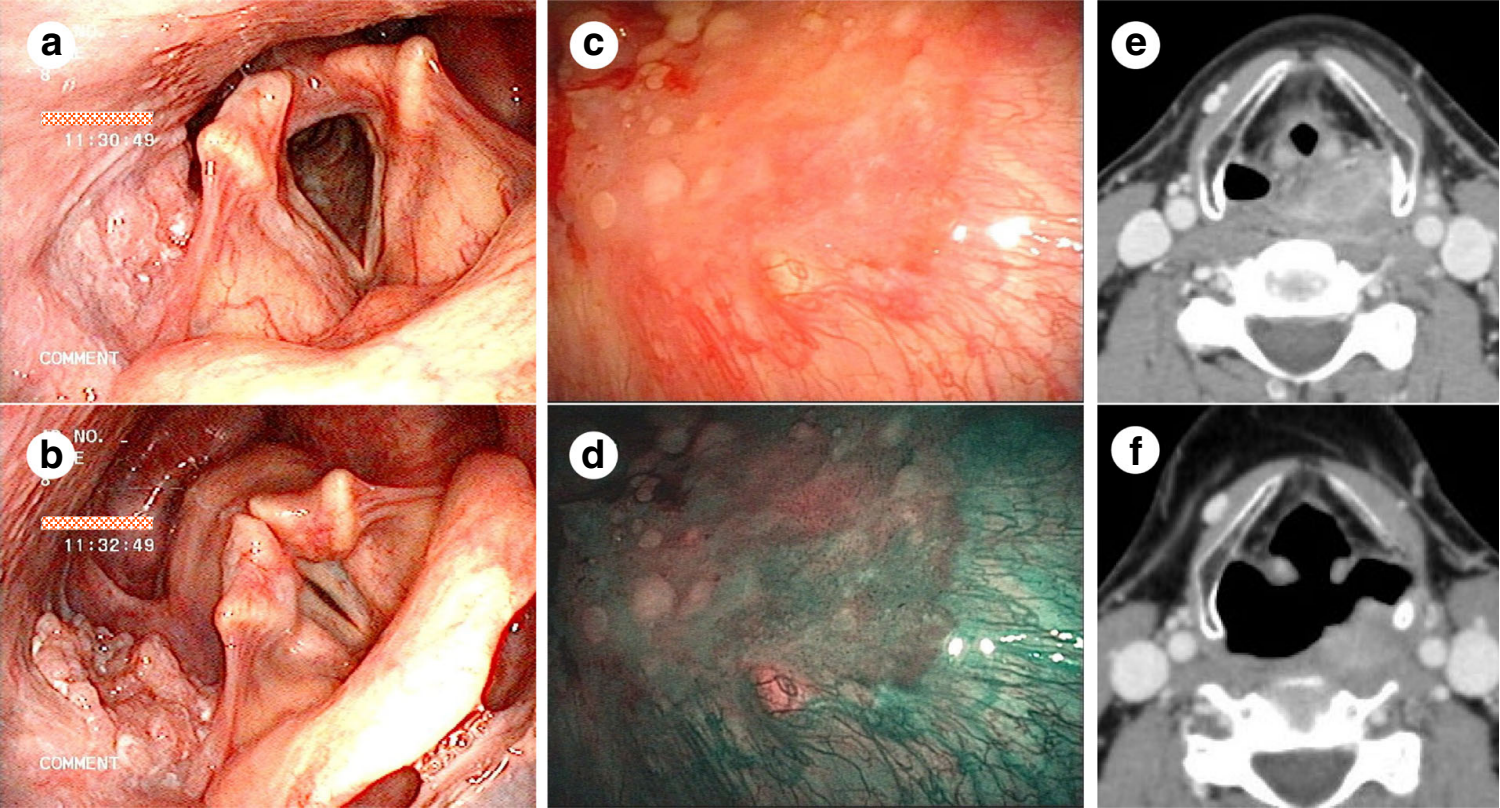
Pre-therapeutic evaluation for TOVS. **a** A transnasal endoscopic view of the larynx and hypopharynx with a tumor on the right pyriform sinus. **b** A view in the same case as **a** under Valsalva maneuver, by which an expanded hypopharyngeal lumen can be observed down to the esophageal entrance. **c** A transnasal endoscopic view of a superficial tumor on the posterior wall of the hypopharynx. **d** A view in the same case as **c** using narrow band imaging, by which loss of typical intra-epithelial papillary capillary loop (IPCL) can be visualized as a brownish area. **e** A normal CT image of the case with an exophytic tumor on the left side of the hypopharyngeal wall. **f** A CT image of the same case as **e** under Valsalva maneuver, by which a tumor can be delineated more clearly in an expanded hypopharyngeal lumen

In the pre-therapeutic evaluation, transnasal endoscopic observation is performed routinely with Valsalva maneuver and head torsion to gain a maximally expanded intraluminal view of the hypopharynx [35–37]. This method enables accurate visualization of tumor extension on the mucosal surface and detailed inspection of the hypopharynx for any other possible lesion down to the esophageal entrance (Fig. 1a and b). Simultaneously, morphological changes in intramucosal microvascular structure (so-called “intra-epithelial papillary capillary loop (IPCL)”) are observed using the narrow band imaging (NBI) mode, an image-enhancing technique equipped in the flexible endoscope ENF-VT2/VQ/VH (Olympus, Japan), to screen for intraepithelial cancer (carcinoma in situ [CIS]) in which loss of typical IPCL can be visualized as a “brownish area” [38–41] (Fig. 1c and d). The Valsalva maneuver is also incorporated in pre-therapeutic CT scanning, by which the usually collapsed hypopharyngeal lumen can expand maximally, especially in the anteroposterior direction, leading to clearer delineation and size measurement of depth and width of a tumor, especially in an exophytic shape [35, 42] (Fig. 1e, f). These assessments are considered indispensable in decision-making regarding applicability of TOVS.

**Fig. 2:**
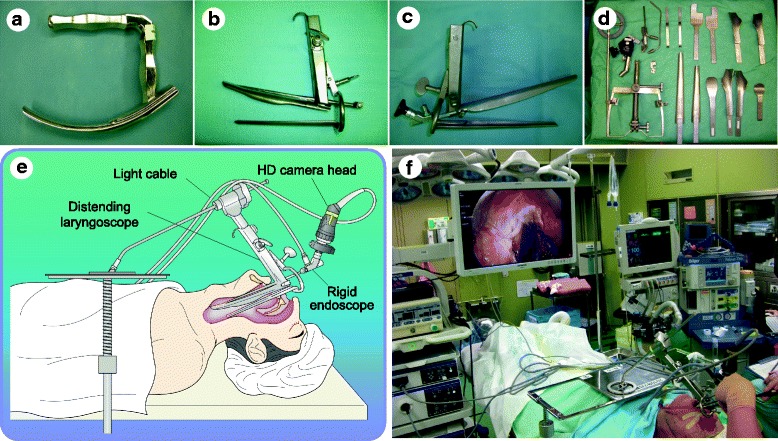
Configurations of TOVS. **a** Curved rigid pharyngolaryngeal blade. **b** Distending laryngoscope. **c** Distending diverticuloscope combined with a rigid endoscope. **d** FK-WO retractor system and its set of various blades. **e** Schematic appearance of the TOVS setting. **f** General scene of the TOVS setting in an operation room. A surgeon at the patient’s head performs surgery by direct bimanual handling of the straight-form surgical instruments and devices while viewing the monitor

Under general anesthesia, thorough inspection is first performed routinely using the aforementioned flexible endoscope with NBI mode and mucosal staining with 1.5% iodine solution that allow visualization of the CIS as an unstained area. For this purpose, laryngeal elevation using a curved rigid pharyngolaryngeal blade (Fig. 2a) (Nagashima Medical Instruments, Japan) is helpful in keeping the hypopharynx expanded, thus providing a favorable view of the entire pharyngolaryngeal lumen, although its benefit is limited to a flexible endoscope [43]. In this step, the exact resection line can be determined based on both the mucosal extent visualized by iodine staining and submucosal extent estimated by evaluating tumor mobility through direct palpation using forceps.

### Surgical procedures

To provide a straight surgical view with broad working space for TOVS, the pharyngolaryngeal lumen is kept expanded using a Weerda distending laryngoscope (Fig. 2b) (8858BV, 17 cm in length of the upper spatula, Karl Storz, Germany), distending diverticuloscope (Fig. 2c) (12067 V, 24 cm in length of the upper spatula, Karl Storz), or FK-WO retractor system (Fig. 2d) (Olympus), of which the appropriate position is determined depending on the tumor location and size. A rigid endoscope (videolaryngoscope) 4 mm in diameter (8575AV, 17 cm in length, 15 degree; or 12067VA, 24 cm in length, 0 degree; Karl Storz) connected to an HD camera (OTV-S7ProH-HD-L08E, OTV-S7ProH-HD-12E, or CH-S190-XZ-E; Olympus) is inserted, either by being attached to the distending scope or manually by a surgical assistant, to display an optimal surgical field on a monitor (Fig. 2e, f).

**Fig. 3:**
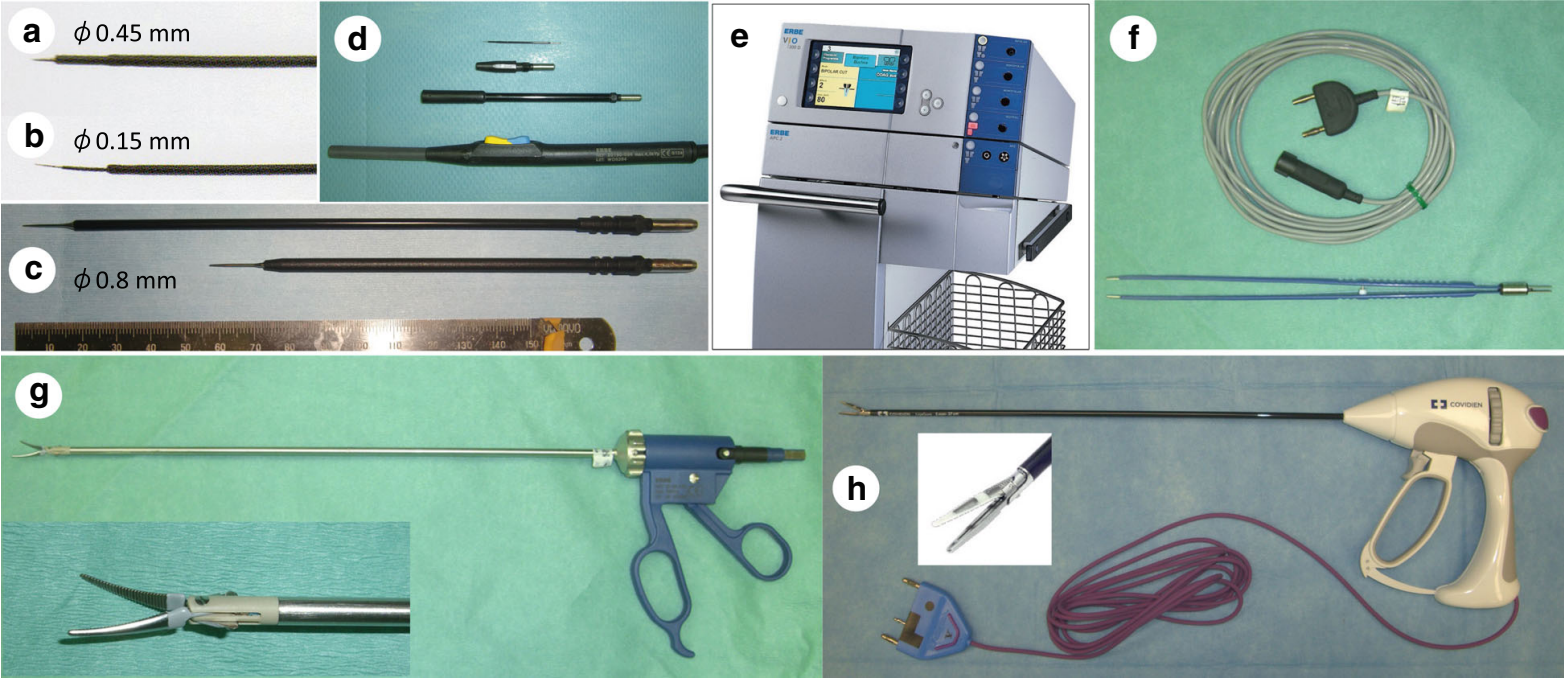
Electrocautery instruments employed in TOVS. **a** Fine needle electrode with a 0.45-mm tip diameter. **b** Fine needle electrode with a 0.15-mm tip diameter. **c** Fine needle electrode with a 0.8-mm tip-shaft diameter. **d** Slim-line hand switch system. **e** Electrosurgical generator VIO300D. **f** Super long bipolar forceps 30 cm in length. **g** BiClamp LAP forceps Maryland type. **h** LigaSure Dolphin Tip

After a tumor’s boundary is confirmed by iodine staining, marking dots on the mucosa are made on the circumference of the lesion with a safety margin ≥5 mm, using a fine needle electrode with tip diameter of 0.45 mm (Fig. 3a) (No.20191-084, Erbe, Germany), tip diameter of 0.15 mm (Fig. 3b) (No.20191-083, Erbe), or tip-shaft diameter of 0.8 mm (Fig. 3c) (No.21191-020 or 21191-070, Erbe) attached to a slim-line hand switch system (Fig. 3d) (No.20190-095, Erbe), in the Soft Coag mode of an electrosurgical generator VIO300D (Fig. 3e) (Erbe). A mixed solution consisting of sodium hyaluronate (MucoUp; Johnson & Johnson K.K., Japan), epinephrine, physiological saline, and indigocarmine is injected through the 25G (gauge) laryngeal fine needle (length, 28 cm) (Nagashima) into the layer beneath the lesion to expand a safety cushion vertically by lifting up the lesion. Before use of electrocautery, a Nelaton soft catheter (12-14 Fr in size) with several additional small holes bored at its tip is inserted transnasally, and the tip is placed just ahead of a the endoscope tip, so that the catheter can evacuate vapor efficiently, which maintains a clear endoscopic view during surgery.

After the mucosa around the marking dots is incised circumferentially with a fine needle electrode in Dry Cut mode, the entire lesion is dissected step-by-step using the same electrode in Dry Cut, Auto Cut, or Swift Coag mode until en bloc resection is accomplished. During the procedure, a surgeon bimanually handles a variety of ready-made straight-form surgical instruments and devices, which enables adequate counter-traction by grasping a margin of the lesion using forceps with one hand, while the other hand manipulates another instrument such as a needle electrode, suction tube, or hemostatic device.

Bleeding points and/or exposed vessels are efficiently coagulated using a super long bipolar forceps 30 cm in length (Fig. 3f) (No.20195-109, Erbe). In case hemorrhage is uncontrollable with the aforementioned method or a bulky tumor can be hauled up from the constrictor muscle, BiClamp LAP forceps Maryland type (Fig. 3g) (No.20195-146, Erbe) and/or LigaSure Dolphin Tip (Fig. 3h) (LS1500, Covidien, USA) are applied to exert more powerful hemostasis. After tumor resection and thorough hemostasis are completed, triamcinolone acetonide solution (Kenacort; 40 mg/mL; Bristol-Meyers Squibb, Japan) is injected evenly into the residual submucosal layer of the wound to prevent postoperative edema and excessive scar formation resulting in stricture [44, 45].

In patients diagnosed as clinically lymph node metastasis-positive, neck dissection was performed as an initial treatment basically on the same day in most patients. In some patients, in whom the resectability of the primary tumor by TOVS was not predictable, neck dissection was performed at a later date after a completeness of tumor resection was pathologically confirmed. On the other hand, in case the resectability of the neck lesion was unpredictable, neck dissection was performed first and was followed by TOVS after a pathological curability of the neck lesion was ascertained.

**Fig. 4:**
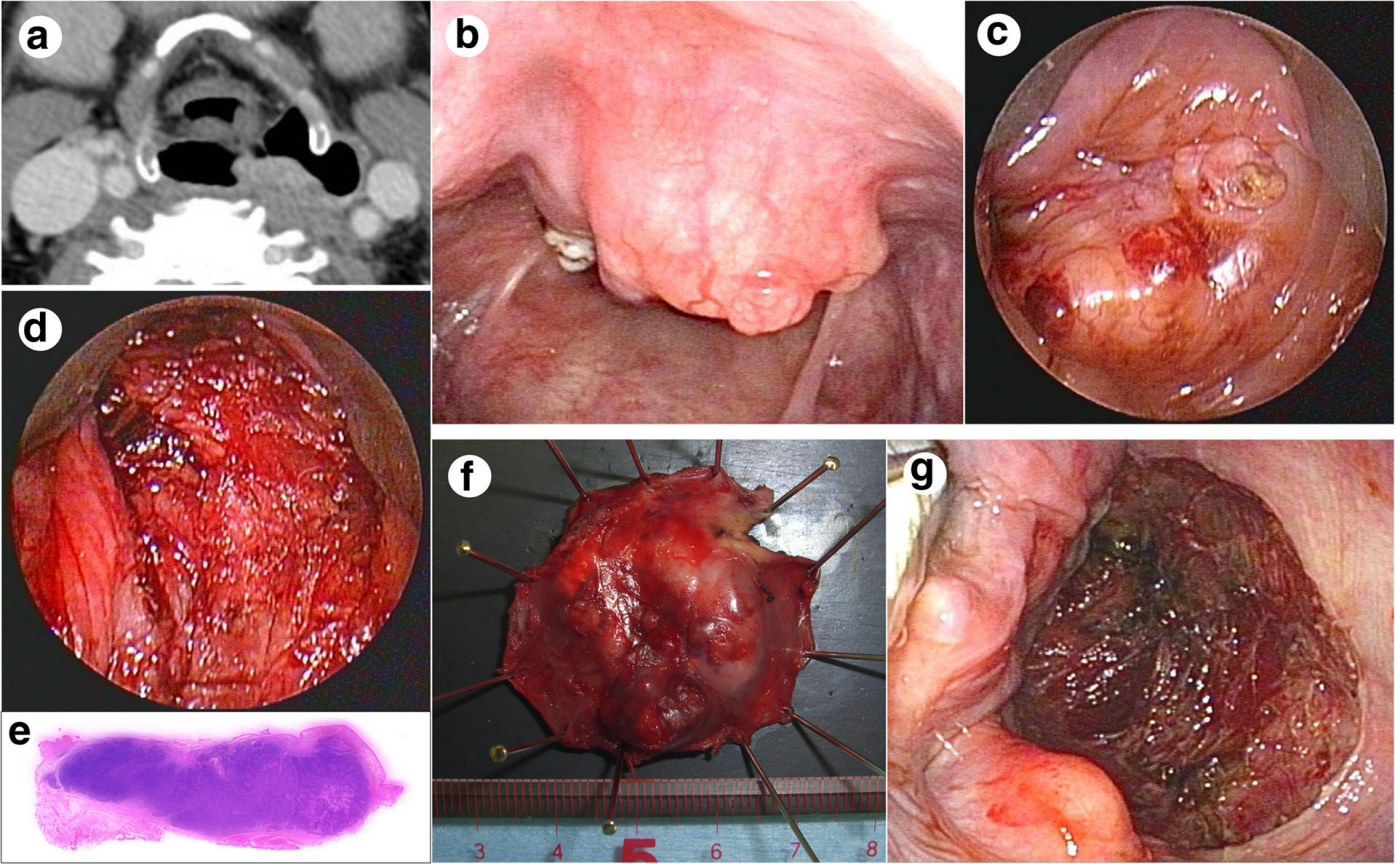
A case in which TOVS was performed for a tumor on the posterior wall. **a** CT image under Valsalva maneuver showing a T2 tumor on the posterior wall of the hypopharynx. **b** Transnasal endoscopic view of the tumor under Valsalva maneuver. **c** Endoscopic view of the tumor just before resection. **d** Endoscopic view of the wound just after resection. **e** Section of the tumor specimen stained with hematoxylin and eosin. **f** Macroscopic view of the tumor specimen resected. **g** Transnasal endoscopic view of the wound just after thorough hemostasis. Inferior pharyngeal constrictor muscle was widely exposed

**Fig. 5:**
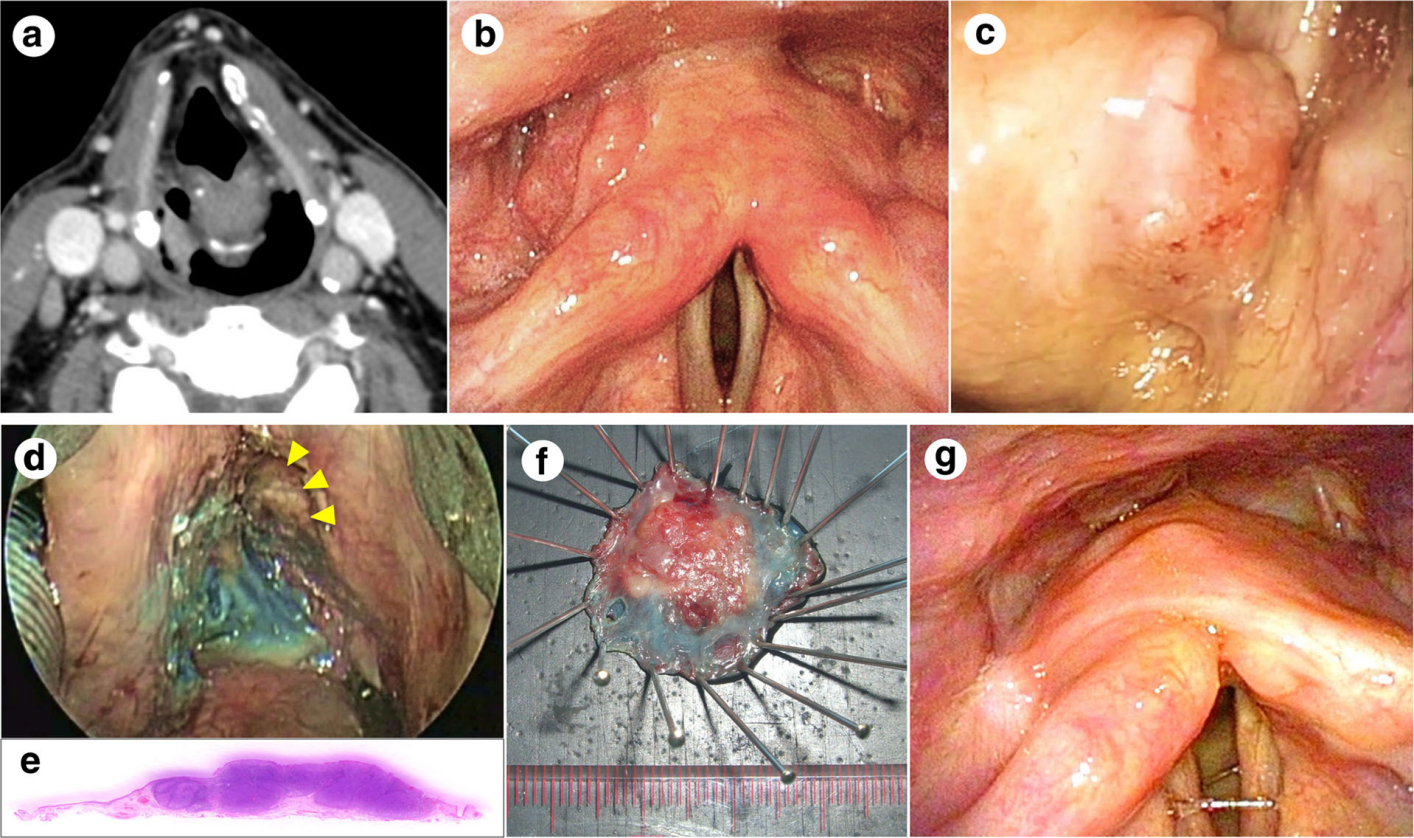
A case in which TOVS was performed for a tumor on the pyriform sinus. **a** CT image under Valsalva maneuver showing a T1 tumor on the right pyriform sinus of the hypopharynx. **b** Transnasal endoscopic view of the tumor under Valsalva maneuver. **c** Endoscopic view of the tumor just before resection. **d** Endoscopic view of the wound just after resection. Thyroid cartilage was partially exposed (arrow heads). **e** Section of the tumor specimen stained with hematoxylin and eosin. **f** Macroscopic view of the tumor specimen resected. **g** Transnasal endoscopic view of the hypopharynx 3 months after resection

Representative cases in which TOVS was performed are presented in Figs. 4 and 5.

### Adjuvant treatments

Regarding the surgical margin in the final histopathology, if the horizontal margin was undoubtedly positive, reoperation of TOVS was considered. If the vertical margin was obviously positive despite a curative intent, the patients underwent open PPL and were excluded from the study.

Concerning pathologically positive lymph node metastasis, if pathological N (pN)-stage was pN0, pN1, or pN2a, we held to a strict observation policy. In patients with pN2b or more, if the number of positive nodes was more than three, positive nodes were distributed in more than one level, or extracapsular spread was revealed, adjuvant cis-platinum (CDDP)-based CCRT was administered. Otherwise, we retained strict observation.

### Patient population

From April 2007 to March 2014, 85 patients with HPC or SGC who met the aforementioned criteria underwent TOVS with or without neck dissection at the Department of Otorhinolaryngology–Head and Neck Surgery, Keio University Hospital (Tokyo, Japan). Among them, patients who subsequently underwent open PPL due to positive vertical margin (*n* = 4), those whose tumor was residual or recurrent after an initial treatment elsewhere (*n* = 3), those treated without a curative intent (*n* = 2), those with simultaneous distant metastasis (*n* = 2), and those with non-SCC malignancy (*n* = 2) were excluded from the study. The remaining 72 patients, who had a minimum follow-up period of 24 months or until the patient’s death, were considered eligible for inclusion in this cohort.

Detailed clinical data of the patients were retrieved from the database. Treatment outcomes were analyzed to evaluate the clinical validity of TOVS as a surgical organ preservation strategy.

### Outcome measures and statistical analysis

All survival probabilities were estimated by using the Kaplan-Meier method. Cause-specific survival (CSS, events: death due to the disease [any of TNM]), overall survival (OS, events: all death), larynx-preserved CSS (LP-CSS, events: total laryngectomy, total pharyngolaryngectomy, or TNM-related death), and loco-regional controlled CSS (LRC-CSS, events: local or regional relapse, or TNM-related death) were analyzed as oncological endpoints.

The generalized Wilcoxon test and the univariate Cox proportional hazards model were used to examine the significance of differences in survival outcomes associated with patient/disease characteristics, including age, sex, tumor site, T stage, N stage, existence of multiple cancers, and history of radiation on the neck. The estimated hazard ratio (HR) and 95% confidence interval (CI) were calculated. The multivariate Cox proportional hazards model further assessed independent significance of the aforementioned variables without sequential and/or stepwise variable selection. *P* values <0.05 were considered statistically significant. All statistical analyses were performed using EXCEL Multivariate Analyses for MAC Ver. 3.0 (Esumi Co., Ltd., Tokyo).

## Results

### Patient characteristics

Demographic and disease characteristics of the 72 patients, including age, sex, primary tumor site, T stage, N stage, and disease stage, are summarized in Table 1. Notably, while 37 patients belonged to N0 (51.4%), the remaining 35 patients (48.6%) had lymph node metastasis. Regarding disease stage distribution, one-third of the patients (*n* = 24, 33.3%) were stage IV. Furthermore, 54 patients (75.0%) had multiple cancers in the head and neck region, other regions, or both; as well as synchronously, metachronously, or both, by the time of the last follow-up. Among them, 12 patients (16.7%) had a history of radiation on the neck for other previous cancers.

**Table 1:** Patient characteristics (*n* = 72)

Characteristics		No.	%
Age, y			
	Median (range)	68 (46-88)	
	Mean ± SD	66 ± 9	
Sex			
	Men	67	93.1
	Women	5	6.9
Tumor site			
	Hypopharynx	58	80.6
	Supraglottis	14	19.4
T stage			
	Tis	9	12.5
	T1	23	31.9
	T2	33	45.8
	T3	7	9.7
N stage			
	N0	37	51.4
	N1	11	15.3
	N2a	1	1.4
	N2b	18	25.0
	N2c	4	5.6
	N3	1	1.4
Stage			
	0	9	12.5
	I	14	19.4
	II	13	18.1
	III	12	16.7
	IVA	23	31.9
	IVB	1	1.4
Multiple cancer			
	No	18	25.0
	Yes	54	75.0
Previous RT on the neck			
	No	60	83.3
	Yes	12	16.7

*SD* Standard deviation, *RT* Radiotherapy

### Surgical results and additional treatments

As summarized in Table 2, we achieved en bloc tumor resection by TOVS in 66 patients. On the other hand, blockwise resection was necessary in the remaining six patients due to relatively wider and/or deeper lesions, including three patients with a T3 invasive tumor that spread over the arytenoid, pyriform sinus, and postcricoid; two with a T2 tumor that extended to the cervical esophagus; and one with a T2 superficial tumor that spread across a semi-circumference of the hypopharynx just above the esophageal entrance. However, such blockwise resections in all these patients were performed in the first 3 years when the surgeons had relatively less experience, but they were not performed afterward.

**Table 2:** Surgical results and additional treatments (*n* = 72)

Outcomes		No.	%
Primary resection			
	En bloc	66	91.7
	Blockwise	6	8.3
Neck dissection			
	No	36	50.0
	Unilateral	32	44.4
	Bilateral	4	5.6
Additional RT			
	No	56	77.8
	Adjuvant	12	16.7
	Secondary	4	5.6

Regarding the surgical margin status, obviously positive horizontal margin was not found in the final histopathology of any patient who underwent TOVS with a curative intent. This is thought to be a result of appropriate confirmation of the tumor’s boundary on the mucosal surface, sufficient additional resection in case the margin was suspected to be positive, and in part with the help of abovementioned blockwise resection in case en bloc resection was impossible. On the other hand, positive vertical margin was observed in seven of the 13 patients who underwent TOVS but were excluded from the study, which included four patients who subsequently underwent open PPL, two patients treated without a curative intent, and one patient with pharyngeal synovial sarcoma who also subsequently underwent open PPL. All cases of incomplete resection due to the positive vertical margin occurred in the first 2 years when the surgeons’ expertise was likely insufficient.

Neck dissections were performed as an initial treatment in 36 patients (50.0%), in which 32 patients were unilateral and four were bilateral, for therapeutic purposes based on clinical N stage. Regarding the timing of neck dissection, it was done on the same day as TOVS in 26 patients, at a later date within 2 weeks after TOVS in seven patients, and within 3 weeks before TOVS in three patients. All but one patient (i.e., *n* = 35, 48.6%) were pathologically positive in the lymph node. Among them, two patients additionally underwent neck dissection on the contralateral side due to delayed neck metastasis that developed in the untreated side.

Postoperative CDDP-based CCRT (50-66 Gy) was administered to 12 patients as adjuvant therapy, including nine patients with N2b, two with N2c, and one with N3. The reasons for adjuvant CCRT were extracapsular spread (*n* = 2), more than three positive lymph nodes (*n* = 2), or both (*n* = 5); or were very close to or had an equivocal surgical margin at the primary site (*n* = 3). Furthermore, another four patients who belonged to N2a (*n* = 1) or N2b (*n* = 3) and repeatedly developed multiple second primary cancers in the pharyngolaryngeal region ultimately underwent CCRT (*n* = 1), RT plus weekly cetuximab (*n* = 1), or RT alone (*n* = 2). Thus, in total, RT was administered to 16 patients (22.2%). Since other 12 patients had a history of radiation on the neck, the remaining 44 patients (61.1%) were spared from RT during the follow-up period.

Regarding 17 patients who underwent additional surgery for second or later primary tumor in the pharyngolaryngeal region, TOVS was repeated in 12 patients with relatively smaller tumors, open PPL was applied to three patients with relatively larger tumors, and the other two patients who had a history of previous RT on the neck ultimately underwent total laryngectomy as salvage therapy.

### Surgical complications

Complications related to TOVS are summarized in Table 3. The endotracheal tube was removed in an operation room after the surgery regardless of additional neck dissection in most patients (*n* = 70). In only two patients who developed laryngopharyngeal edema following a blockwise resection of T3 tumor associated with a unilateral neck dissection, a transient tracheostomy was placed before extubation and closed within a week. No patient required prolonged mechanical ventilation or an intensive care unit stay postoperatively.

**Table 3:** Complication and dysfunction (*n* = 72)

Category		No.	%
Complication			
Respiration-related			
	Temporary tracheostomy	2	2.8
	Prolonged mechanical ventilation	0	0.0
Surgical site-related			
	Pharyngeal fistula	2	2.8
	Subcutaneous emphysema	4	5.6
Dysfunction			
Swallowing-related			
	Nasogastric tube placement	16	22.2
	Preventive balloon dilation	3	4.2
	Gastrostomy tube placement	0	0.0
	Aspiration pneumonia	2	2.8
	Persistent dysphasia	3	4.2
Phonation-related			
	Permanent vocal dysfunction	0	0.0

A pharyngeal fistula formed in two patients who underwent resection of tumor on the pyriform sinus followed by an ipsilateral neck dissection. In the first case, a fistula was noticed just after extubation because of continuous leakage of expiratory air into the drainage tube placed under the neck skin, so it was located immediately in reoperation and closed by suturing mucosal layers with the sternohyoid muscle. In the second case, a fistula was found during an extended neck dissection for N3. Although the fistula was closed cautiously during surgery and drainage tubes were extracted uneventfully, a small subcutaneous abscess formed in the same position shortly afterward and required local treatments and interruption of oral intake for a week until ultimate closure. No other patients experienced surgical site infections.

Although four patients who did not undergo neck dissection developed cervical subcutaneous emphysema supposedly owing to pharyngeal fissure opened to the surrounding soft tissues, all were absorbed spontaneously. Other minor surgical complications included postoperative minor hemorrhage, partial tooth damage, and injuries of the upper lip.

### Functional results

Postoperative dysfunctions are summarized in Table 3. Fifty-six patients (78%), including all who had Tis or T1 tumors, resumed oral intake on the first or second postoperative day without obvious dysphasia.

In the other 16 patients (22%), all of whom had a T2 or T3 tumor resected, nasogastric feeding tubes were placed for a median of 4 (range: 1–12) days. The indication depended on the extent of estimated risk of postoperative dysphasia owing to various factors, including structural changes in the supraglottis leading to aspiration, narrowed esophageal entrance associated with transient mucosal edema, hypersecretion of mucus discharge, history of previous RT on the neck, and wound pain. Among them, two patients, who had T3N2b SGC and underwent adjuvant CCRT, developed aspiration pneumonia during or after CCRT, although they recovered after conservative treatment in association with swallowing rehabilitation. Other three patients, whose T2 HPC required a resection beyond the esophageal entrance, underwent balloon dilation periodically or irregularly to prevent a progressive stricture for 4 to 12 weeks. No patient underwent gastrostomy tube placement.

Overall, the median time until patients resumed oral intake was 2 days (range 1–8 days) and that until patients could take a soft meal was 5 days (range 1–21 days). Eventually, 69 patients (96%) were able to take normal meals. The remaining three patients, comprised of one patient who developed aspiration pneumonia and needed prolonged swallowing rehabilitation, one patient whose progressive stricture of the esophageal entrance could not be avoided, and another patient with Tis HPC who had a history of RT on the neck for previous oropharyngeal cancer, retained persistent dysphasia, although they did not require additional intervention. No patients complained of vocal dysfunction 1 month after the surgery.

### Oncological outcomes and survival analyses

The median follow-up period of all patients (*n* = 72) and that of the patients alive at the time of the analysis (*n* = 56) were 45 (range, 7–105) and 52 (range, 24–105) months, respectively (Table 4). During follow-up, eight patients (11.1%) died of the index cancer (seven of distant metastasis and one of locoregional recurrence), and eight patients (11.1%) died of other causes. At the last follow-up, 54 patients (75.0%) were alive without the disease (including 12 patients who underwent either salvage surgery or CCRT/RT or both, and remained recurrence-free), and two patients (2.8%) were alive with the disease (both with distant metastasis).

**Table 4:** Follow-up information (*n* = 72)

Median follow-up period		Months (range)	
	of all patients	45 (7-105)	
	of survivors (*n* = 56)	52 (24-105)	
Last status		No.	%
	NED	54	75.0
	AWD	2	2.8
	DOD	8	11.1
	DOC	8	11.1

*NED* No evidence of the disease, *AWD* Alive with the disease, *DOD* Died of the disease, *DOC* Died of other causes

**Fig. 6:**
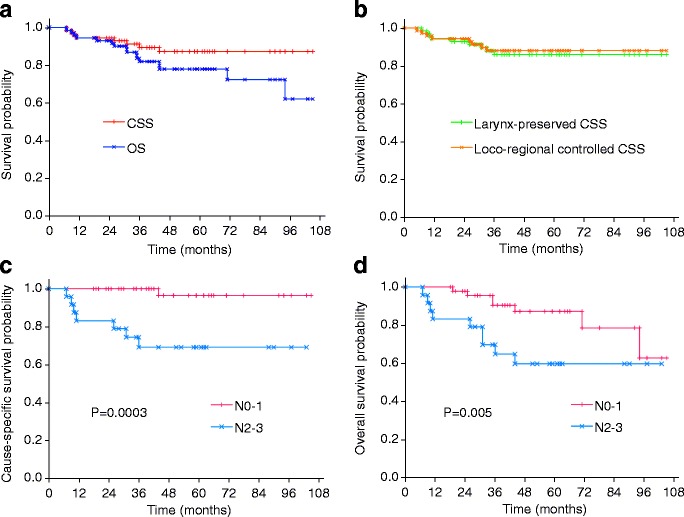
Kaplan-Meier survival curves. **a** Cause-specific survival (CSS, *red*) and overall survival (OS, *blue*) of all patients (*n* = 72). The 5-year CSS and OS rates were 87.3 and 77.9%, respectively. **b** Larynx-preserved CSS (LP-CSS, *green*) and loco-regional controlled CSS (LRC-CSS, *orange*) of all patients. The 5-year LP-CSS and LRC-CSS rates were 86.0 and 88.0%, respectively. **c** CSS according to N stage (N0-1 [*n* = 48] vs N2-3 [*n* = 24]). The 5-year CSS rates were 96.4% for N0-1 (*pink*) and 69.2% for N2-3 (*light blue*) (generalized Wilcoxon test, *P* = 0.0003). **d** OS according to N stage. The 5-year OS rates were 87.3% for N0-1 (*pink*) and 59.9% for N2-3 (*light blue*) (generalized Wilcoxon test, *P* = 0.005)

The 3-year CSS and OS rates were 89.4% (95% CI, 82.0–96.9%) and 81.9% (95% CI, 72.6–91.2%), respectively (Fig. 6a). The 3-year LP-CSS and LRC-CSS rates were 86.0% (95% CI, 77.4–94.6%) and 88.0% (95% CI, 80.2–95.9%), respectively (Fig. 6b). Furthermore, 5-year CSS and OS rates were 87.3% (95% CI, 78.8–95.7%) and 77.9% (95% CI, 67.5–88.3%), respectively, whereas the 5-year LP-CSS and LRC-CSS rates remained the same as those of the 3-year rates, respectively. Because the cohort included nine patients with Tis lesions who inevitably raise the survival rates, each endpoint was also evaluated for the remaining 63 patients; 5-year CSS, OS, LP-CSS, and LRC-CSS rates were 81.4%, 76.6%, 80.5%, and 81.2%, respectively. However, these results were not significantly worse than those described above.

The results of the Cox proportional hazards model analysis are summarized in Table 5. In univariate analysis, patients with N2-3 showed significantly worse CSS (*P* = 0.010) and OS (*P* = 0.032) than those with N0-1, whereas no other factor was significantly associated with CSS or OS. Kaplan-Meier survival curves according to N stage with generalized Wilcoxon tests are shown in Fig. 6c and d. The 5-year CSS rates were 96.4% (95% CI: 89.6–100.0) for N0-1 and 69.2% (95% CI: 50.0–88.4) for N2-3 (*P* = 0.0003, Fig. 6c), whereas the 5-year OS rates were 87.3% (95% CI: 76.8–97.9) for N0-1 and 59.9% (95% CI: 39.3–80.4) for N2-3 (*P* = 0.005, Fig. 6d). Multivariate analysis using the Cox proportional hazards model revealed independent significance of N2-3 as an unfavorable prognostic factor in both CSS (HR = 25.51 [95% CI: 2.29–284.17] vs. N0-1, *P* = 0.008) and OS (HR = 4.90 [95% CI: 1.26–19.08] vs. N0-1, *P* = 0.022) (Table 5).

**Table 5:** Univariate and multivariate Cox regression analyses for cause-specific survival and overall survival (*n* = 72)

Variables			Cause-specific survival						Overall survival					
		No.	Univariate analysis			Multivariate analysis			Univariate analysis			Multivariate analysis		
			HR	(95% CI)	*P*-values	HR	(95% CI)	*P*-values	HR	(95% CI)	*P*-values	HR	(95% CI)	*P*-values
Age, y														
	<70	45	1.00	reference		1.00	reference		1.00	reference		1.00	reference	
	≧70	27	0.93	(0.22-3.89)	0.920	1.15	(0.24-5.42)	0.858	0.94	(0.34-2.60)	0.913	1.00	(0.34-2.89)	0.994
Sex														
	Men	67	1.00			1.00			1.00	reference		1.00	reference	
	Women	5	not calculable^a^		−	not calculable^a^		−	0.61	(0.08-4.75)	0.636	1.27	(0.13-12.38)	0.835
Tumor site														
	HPC	58	1.00	reference		1.00	reference		1.00	reference		1.00	reference	
	SGC	14	0.57	(0.07-4.68)	0.605	0.38	(0.04-3.43)	0.386	0.60	(0.14-2.64)	0.498	0.54	(0.11-2.69)	0.450
T stage														
	Tis + T1	32	1.00	reference		1.00	reference		1.00	reference		1.00	reference	
	T2-3	40	2.69	(0.54-13.34)	0.227	0.68	(0.09-5.24)	0.714	1.58	(0.57-4.35)	0.380	1.27	(0.29-5.54)	0.749
N stage														
	N0-1	48	1.00	reference		1.00	reference		1.00	reference		1.00	reference	
	N2-3	24	15.45	(1.90-125.69)	0.010*	25.51	(2.29-284.17)	0.008*	2.94	(1.09-7.90)	0.032*	4.90	(1.26-19.08)	0.022*
Multiple cancer														
	No	18	1.00	reference		1.00	reference		1.00	reference		1.00	reference	
	Yes	54	1.08	(0.22-5.36)	0.923	2.63	(0.45-15.20)	0.281	1.21	(0.39-3.82)	0.742	1.74	(0.47-6.41)	0.405
Previous RT														
	No	60	1.00	reference		1.00			1.00	reference		1.00	reference	
	Yes	12	0.68	(0.08-5.52)	0.717	not calculable^b^		−	1.09	(0.31-3.83)	0.894	2.23	(0.34-14.86)	0.406

*HPC* hypopharyngeal cancer, *SGC* supraglottic cancer, *RT* Radiotherapy, *HR* hazard ratio, *CI* confidence interval

* Statistically significant (*p* < 0.05)

^a^HR was not calculable because no woman died of the disease

^b^HR was not calculable because of strong confounding with N stage

## Discussion

The present study revealed that the cohort of patients with HPC and SGC who underwent TOVS as an initial treatment according to our criteria had favorable oncological outcomes, even after a long-term follow-up period. Notably, those results were achieved with fairly low incidence of surgical complication and minimal postoperative dysfunction. Thus, we regard TOVS, in combination with neck dissection and adjuvant CCRT if necessary, as one of the excellent therapeutic strategies in terms of organ-function preservation for patients with HPC and SGC.

Although several transoral approaches as less invasive surgery than conventional open PPL have been developed so far, TOVS has its own advantages over other approaches in terms of practical usefulness. When compared with TLM [16–22], TOVS has several technical advantages. First, since an endoscope lens possesses much longer depth range of focus (e.g., from 3 to 30 mm for a lens 4 mm in diameter) and wider angle of view (e.g., >110° for an above-mentioned lens) than that of a microscope, TOVS can provide a much broader surgical view in both horizontal and vertical directions compared to TLM, which helps improve recognition of anatomical orientation. Second, the field of endoscopic view is free from visual restriction due to the inner wall of the laryngeal blade that often restricts the microscopic view. Moreover, manipulation of surgical instruments is not restricted by a microscope interposed between patient and surgeon. Third, en bloc resection of primary tumor achieved by TOVS enables accurate evaluation of pathological findings, especially about margin status, tumor depth, and horizontal diameter, which cannot be assessed in tumor specimens resected blockwise by TLM. Such pathological information, together with differentiation, vascular invasion, and lymphatic invasion, are indispensable not only for judging completeness of resection but also for assessing risk of delayed neck metastasis in clinically N0 patients [46]—this underscores the importance of en bloc resection in decision making regarding additional intervention. Fourth, the NBI mode equipped in endoscopes, including the ENDOEYE FLEX LTF-S190-5 (Olympus), is available even during surgery if necessary [33].

Although indications for use of TORS using da Vinci surgical systems have recently been extended to HPC and SGC in several countries [23–30], TORS has not been approved yet in many countries, including Japan. Instead, TOVS has been developed as a non-robotic transoral surgery in Japan. In comparison with TORS, TOVS has more practical advantages. First, since the surgeon bimanually manipulates surgical instruments directly on the real lesion, unlike TORS, the surgeon can recognize tactile sensations through those instruments; this is essential for assessing tumor invasion to the surrounding tissues and adding adequate counter-traction during the dissection procedure. Second, the cost-effectiveness of TOVS is far higher than that of TORS, because neither an extremely expensive surgical robot nor high-priced disposable equipment is required. Whereas most hospitals still cannot afford da Vinci surgical systems for TORS, the initial cost to introduce TOVS and its running costs are much lower, because most surgical instruments and devices are reusable, not originally designed for TOVS, and can be shared among other surgeries. Accordingly, TOVS can be introduced more easily than TORS in more hospitals in more countries.

Since endoscopes used in TOVS are not yet equipped with binocular vision, a three-dimensional view is not available. However, this does not really affect surgical performance because most otorhinolaryngologists/head and neck surgeons are already familiar with the two-dimensional endoscopic view. Fortunately, such a possible disadvantage compared to TLM and TORS is well compensated for by the use of high-resolution cameras. Furthermore, three-dimensional rigid endoscopes will be introduced in the near future, if necessary.

Besides TLM and TORS, a few other transoral approaches using a flexible gastrointestinal endoscope for early HPC were also reported with favorable outcomes from Japan: endoscopic mucosal resection (EMR) and endoscopic submucosal dissection (ESD) performed by gastroenterologists [47–50] and endoscopic laryngopharyngeal surgery performed by otorhinolaryngologists and gastroenterologists [43]. However, indication for use of these methods was confined to only patients with superficial lesions in the pharynx without lymph node metastasis (N0), excluding patients with SGC, invasive cancer, or lymph node metastasis (≥N1). Thus, distributions of disease stage in these patients, most of whom are at an incipient stage, are largely different from those of TOVS and others. In other words, indication for TOVS is very broad and ranges from superficial, small, or thin lesions (Tis) to invasive, exophytic, or bulky masses (up to T3 defined by size criteria). Notably, such wide-ranging lesions can be resected in the common setting with the same instruments; thus, high versatility is another advantage of TOVS.

A fair comparison of clinical outcomes between TOVS and other surgical approaches such as TLM or TORS seems difficult, because distributions of the disease stage, follow-up periods, and endpoint settings differ among them. However, oncological and functional outcomes of TOVS, including the previous report [33], are mostly comparable to those of TLM and TORS [16–30]. Considering the distribution of disease stage in the patients in our study, a half of them were stage III–IV, the long-term oncological and functional outcomes appeared to be satisfactory. These results may corroborate an overall validity of this therapeutic strategy, including the criteria of indication, the principles of surgical management, and the standards of adjuvant treatments, especially about the relatively low necessity of postoperative RT.

However, it should be noted that strict observation in the follow-up period is another crucial prerequisite to achieve high LP-CSS and LRC-CSS in this cohort, because a majority of them possessed a high risk of developing second primary cancer in the pharyngolaryngeal region, even though the primary lesion was completely resected. In our cohort, although 18 patients developed one or multiple second primary cancer in the pharyngolaryngeal region, all but one was diagnosed at an early stage. Among them, 16 patients were able to preserve the larynx by repeated TOVS alone (*n* = 10), by following TOVS with RT for a third primary tumor with (*n* = 1) or without (*n* = 1) cetuximab, by open PPL alone (*n* = 2), by following open PPL with CCRT for a close margin (*n* = 1), or by RT alone (*n* = 1); in contrast, the other two patients ultimately required total laryngectomy for unavoidable reasons. Therefore, close attention must be maintained throughout follow-up so that second or later primary lesion can be treated appropriately as early as possible.

In both CSS and OS, advanced N stage (N2-3) was found to be the only independent unfavorable prognostic factor in this cohort, probably in part because of relatively low statistical power due to small sample size. Intriguingly, in accordance with the LRC-CSS rate as high as 88.0% at 3 and 5 years, uncontrolled regional failure and related death occurred in only one of 10 patients who developed treatment failure. The remaining nine patients developed distant metastasis without locoregional failure, seven of whom died as a consequence, suggesting that N2-3 is a strong predictor of death due to delayed distant metastasis. In agreement with these results, N2-3 was also found to be an independent unfavorable predictor of distant metastasis-free survival (data not shown). Thus, in common with many other cancers at an advanced stage, the most critical unsolved issue appears to be management of distant metastasis, irrespective of differences in therapeutic modality for locoregional lesions.

Regarding postoperative management-related issues, the incidence of temporary tracheostomy is relatively low in cohorts who underwent TOVS, including both the present (2.8%) and previous (6.7%) reports [33], while it varies largely in each of the other surgical approaches such as TLM, TORS, and ESD/EMR. Several recent studies reported a relatively high incidence of tracheostomy in TLM (12.2–16.0%) [21, 22], TORS (23.8–100%) [26, 29], and ESD/EMR (16.3%) [49]; however, these numbers seem to reflect the prophylactic use of tracheostomy to a certain extent. On the other hand, in some studies in which no patients underwent tracheostomy, instead, a high incidence of prolonged intubation of more than 24 h was reported in TLM (27.1%) [16], TORS (60.0%) [30], and ESD (30.8%) [47]. Although the necessity of tracheostomy principally depends on the extent of postoperative laryngeal edema based on depth and width of the defect after tumor resection and its location, because such estimation involved personal experience and expertise in airway management, making a decision of tracheostomy is rather subjective. In our experience, careful observation of the surgical site in view of the whole laryngopharynx under endoscope by skilled otorhinolaryngologists just after resection is sufficient to make an appropriate decision.

Fortunately, no patients experienced major postoperative hemorrhage that required return to the operation room for emergency treatment. Since we have been well aware of the potential risk of hemorrhage that can be fatal, maximum attention has always been paid to completeness of hemostasis after resection. In our practice, use of a super long bipolar forceps and/or BiClamp LAP forceps can efficiently control any active bleeding during surgery without difficulty. In case a patient has taken an anticoagulant agent, proper management of the drug during the perioperative period is also imperative to prevent increased hemorrhage.

Although aspiration pneumonia was found only in three patients, slight mucus influx into the glottis was observed temporarily in some other patients, especially at an early period after TOVS, suggesting that silent aspiration might occur more frequently. It can be assumed that such transient aspiration that could develop into pneumonia was preventively resolved in many ways, including minute endoscopic evaluation of swallowing function before and after resuming oral intake, timely removal of feeding tube without delay, thoughtful adjustment of form of meal by a dietitian, and appropriate introduction of swallowing rehabilitation if necessary. In addition, effects of preventive efforts to avoid progressive stricture, such as injection of steroid just after resection and repeated balloon dilation if necessary, also appeared to be reflected by a low incidence of persistent dysphasia. Furthermore, in some patients with advanced N stage who underwent neck dissection, reduced intensity of adjuvant CCRT, which was based on the detailed histopathological finding of lymph node metastasis, was also assumed to partly contribute to better function-preservation.

## Conclusions

TOVS for patients with HPC and SGC as an initial treatment provided favorable long-term oncological outcomes with low frequency of surgical complication and minimal functional impairment, corroborating its validity as a therapeutic strategy for this cohort. While high LP-CSS and LRC-CSS reflected its excellent locoregional control, advanced N stage determined as an independent prognostic factor in both CSS and OS indirectly reflected higher risk of delayed development of distant metastasis as an unsolved issue. Considering its sound clinical outcomes and various practical advantages, TOVS can be a dependable, less invasive, and cost-effective surgical option of an organ-function preservation strategy for HPC and SGC.

## Abbreviations

CCRT: Concurrent chemoradiation
CDDP: Cis-platinum
CI: Confidence interval
CIS: Carcinoma in situ
CRT: Chemoradiation
CSS: Cause-specific survival
EMR: Endoscopic mucosal resection
ESD: Endoscopic submucosal dissection
HPC: Hypopharyngeal cancer
HR: Hazard ratio
IPCL: Intra-epithelial papillary capillary loop
LP: Larynx-preserved
LRC: Loco-regional controlled
OS: Overall survival
PPL: Partial pharyngolaryngectomy
RT: Radiation
SCC: Squamous cell carcinoma
SGC: Supraglottic cancer
TLM: Transoral laser microsurgery
TORS: Transoral robotic surgery
TOVS: Transoral videolaryngoscopic surgery
